# Intranasal Esketamine for Treatment-Resistant Depression in Real-World Multicenter Practice: Clinical Effectiveness, Functional Recovery, and Patient-Reported Quality of Life

**DOI:** 10.1192/j.eurpsy.2026.10612

**Published:** 2026-07-31

**Authors:** A.-I. De Santiago-Diaz, S. Arostegui-Uranga, A. Pérez-Poza, F. A. Rodríguez-Batista, V. Roselló-Molina, F. Mora-Mínguez, L. Gutiérrez-Rojas, E. Sáez-Fuentes, L. Montes-Reula, M. A. Madrazo-Maza

**Affiliations:** 1 Psychiatry, University Hospital Valdecilla, Santander; 2 University Hospital of Donostia, San Sebastián; 3 Psychiatry, University Hospital Miguel Servet, Zaragoza; 4 Psychiatry, University Hospital Doctor Negrín, Las Palmas de Gran Canaria; 5 Psychiatry, University Hospital of Ribera, Alzira; 6 Psychiatry, University Hospital Infanta Leonor, Madrid; 7 Psychiatry, University Hospital San Cecilio, Granada; 8 Hospital of Galdákano, Vizcaya; 9 University Hospital San Jorge, Huesca; 10 University Hospital Basurto, Bilbao, Spain

## Abstract

**Introduction:**

Treatment-resistant depression (TRD) is associated with poor quality of life, functional impairment, and increased healthcare use. Intranasal esketamine (IN-ESK) has demonstrated efficacy and safety in TRD. Patient-reported outcomes (PROMs) and experience measures (PREMs) are essential to assess functional recovery and quality of life.

**Objectives:**

To evaluate the clinical effectiveness, functional impact, and quality of life of intranasal esketamine in people with TRD, integrating the patient experience.

**Methods:**

Retrospective, observational, multicenter study in 10 Spanish hospitals, with the approval of the local ethics committee. Subjects: 149 patients aged ≥18 years with TRD treated with IN-ESK. Assessments: depressive severity (MADRS), functional status (FAST), quality of life (EQ-5D). Outcomes: response (≥50% MADRS reduction), remission (MADRS < 7), functional improvement (FAST), EQ-5D changes. Time points: 4 weeks, 6 months, 12 months. Analysis: means, frequencies, chi-squared tests.

**Results:**

149 patients were included (mean age 50 years, 64% women, 58% living with a partner, 82% living in urban areas, 62% with children, and 42% on sick leave). 60% of patients had recurrent depressive disorder. Mean episode duration 21 months; 46% >1 year. Patients failed a mean of .4 antidepressants (range 2–12). Remission achieved in 41% (14% within four first weeks), response 34%. This represents 75% favourable clinical outcomes. Significant functional improvement was observed (mean FAST reduction 10 points at 4 weeks, >16 points at 6 months, and 17 points at 12 months). EQ-5D scores increased, reflecting improved quality of life (>16 points at 4 weeks, >30 points at 6 months, and 36 points at 12 months), achieving more than a 100% improvement in quality of life at one year compared with baseline before treatment. Remission and functional recovery were higher in episodes <2 years (48% vs 18%, p=0,001). Patients on sick leave improved more if leave ≤6 months (remission 59% vs 33%, p=0,05).

**Conclusions:**

IN-ESK improves depressive symptoms, functioning, and quality of life in TRD. PROMs and PREMs provide complementary insight into effectiveness. Shorter episodes, and brief sick leave were associated with better outcomes. Earlier treatment initiation linked to higher clinical and functional recovery. Further studies are needed to confirm these results.

**Disclosure of Interest:**

A.-I. De Santiago-Diaz Grant / Research support from: Johnson&Johnson, Speakers bureau of: Johnson& Johnson, S. Arostegui-Uranga Speakers bureau of: Johnson&Johnson, A. Pérez-Poza: None Declared, F. Rodríguez-Batista: None Declared, V. Roselló-Molina Speakers bureau of: Johnson&Johnson, F. Mora-Mínguez Speakers bureau of: Johnson&Johnson, L. Gutiérrez-Rojas Speakers bureau of: Johnson&Johnson, E. Sáez-Fuentes Speakers bureau of: Johnson&Johnson, L. Montes-Reula: None Declared, M. Madrazo-Maza Speakers bureau of: Johnson&Johnson.

